# The influence of intraoperative auditory brainstem responses on vibroplasty coupling-quality and analysis of the impact of different fixation steps on the coupling

**DOI:** 10.1007/s00405-023-08103-9

**Published:** 2023-07-13

**Authors:** Daniel Dejaco, David Riedl, Timo Maria Gottfried, Matthias Santer, Annette Runge, Josef Seebacher, Philipp Zelger, Bicego Lia, Schmutzhard Joachim

**Affiliations:** 1 Department of Otorhinolaryngology—Head and Neck Surgery, Medical University of Innsbruck, Anichstr. 35, 6020 Innsbruck, Austria; 2 Department of Medical Psychology, Medical University of Innsbruck, Schöpfstr. 23a, 6020 Innsbruck, Austria; 3 Department for Hearing, Speech and Voice Disorders, Medical University of Innsbruck, Anichstr. 35, 6020 Innsbruck, Austria; 4 MED-EL Elektromedizinische Geräte G.M.B.H., Fürstenweg 77a, 6020 Innsbruck, Austria

**Keywords:** Otology, Mild-to-profound hearing loss, Intraoperative auditory-brainstem-responses, Vibroplasty, Coupling-quality, Outcome

## Abstract

**Purpose:**

The Vibrant Soundbridge (VSB) is an established active-middle-ear-implant for patients with moderate-to-profound hearing-loss. This surgery is referred to as “Vibroplasty”. Sufficient transfer of the VSB’s floating-mass-transducers (FMT) energy to the inner ear is a crucial factor influencing the coupling-quality (CQ). However, assessing CQ is hamper by two issues: the method of CQ-assessment itself and the method of FMT-fixation during Vibroplasty.

**Methods:**

This prospective study explored the influence of intraoperative auditory-brainstem-response (+ ABR) measurements and various fixation methods on postoperative CQ after Vibroplasty as compared to matched-patients after Vibroplasty without intraoperative ABR (-ABR). Propensity-score-matching was performed based on preoperative bone-conduction-pure-tone-average-3 (BC-PTA3) at 1-, 2- and 4 kHz. Primary outcome parameters were postoperative CQ-PTA3, intraoperative ABR threshold for various fixation methods and postoperative BC-PTA3.

**Results:**

A total of 28 patients were included, of which 14 were + ABR. Preoperative BC-PTA3, sex, age, and number of previous surgeries did not differ significantly between groups (all *p* > 0.301). Mean postoperative CQ-PTA3 was significantly better for + ABR (1.8 vs. 12.3 dB-HL; *p* = 0.006). Mean intraoperative ABR threshold was superior for cartilage-counter-bearing and cartilage-housing compared to additional fixation with injectable-platelet-rich- fibrin (53 vs. 56 & 57 dB-HL, respectively; *p* = 0.04; *η*^2^ = 0.33). Mean postoperative BC-PTA3 did not significantly differ between patients (41.4 vs. 41.8 dB-HL; *p* = 0.77). A total of 7% of the patients required intraoperative readjustment of the FMT based on unsatisfactory intraoperative ABR threshold.

**Conclusion:**

Intraoperative ABR measurement resulted in significantly better postoperative CQ. Cartilage-counter-bearing and cartilage-housing were observed to have superior CQ. A total of 7% of the patients could be spared revision-Vibroplasty due to intraoperative ABR measurement.

## Introduction

In patients with moderate to profound sensorineural- (SNHL), conductive- (CHL) or mixed hearing loss (MHL), hearing aids (HAs) are considered the standard treatment for hearing rehabilitation [1]. Occasionally, patients are unable to wear HAs because of medical (i.e., chronic otitis externa) or technical problems (i.e., feedback, sound distortion) [1]. In these patients, active-middle- ear-implants (AMEI) are currently considered an appropriate alternative to HAs [2].

An established device for this type of surgery is the Vibrant Soundbridge® (VSB, MED-EL; Innsbruck, Austria) [3, 4]. The main part of the VSB is the floating-mass-transducer (FMT), which transmits vibratory motion to various structures of the middle ear [3, 4]. Introduced in the 1990s [5], the VSB obtained its original indication for SNHL in 2002 [6], which was expanded to MHL in 2006 [7]. Originally, the FMT was coupled to the long incudal process (LP) [6]. Additional coupling-sites with various advantages, including the round window (RW) [7], oval window (OW) [8, 9] and short incudal process (SP) [10], were proposed, which are referred to as “Vibroplasty”.

For adequate postoperative hearing rehabilitation after Vibroplasty, it is essential that the FMT’s energy is sufficiently transferred to the inner ear. The coupling-quality of the FMT plays a crucial role in this energy transfer [11]. There are different methods to assess postoperative coupling-quality after Vibroplasty including subtracting postoperative BC-PTA4 from Vibrogramm-PTA4 and postoperative BC-PTA3 from Vibrogramm-PTA3 [11]. Due to previously described insufficient amplification of lower frequencies of the VSB, recently the latter method has more frequently used [12]. Cebulla and colleagues were the first to report intraoperative estimation of coupling-quality of the VSB using Acoustic AP [12]. Insufficient coupling-quality has been discussed as one of the main contributors to unsatisfying postoperative hearing rehabilitation than can result in revision surgery [11]. Schraven and colleagues reported a revision rate after AMEIs with FMT technology of up to 15.6% in a prospective single-center cohort study including 83 patients. The authors concluded that optimization of coupling is necessary to achieve better audiological results [11].

FMT coupling is currently hampered by two methodological issues: (1) the assessment of coupling-quality itself and (2) the different methods of fixation of the FMT during Vibroplasty.

Although there is currently no standard for intraoperative assessment of FMT coupling-quality during Vibroplasty, various smaller (mainly retrospective) studies have been published on this topic. Verhaegen and co-authors retrospectively explored auditory steady state responses (ASSR) in RW- and OW-Vibroplasty in four adult patients with MHL. They reported ASSR threshold to be a good method to determine the FMT’s optimal position [14]. Radeloff and co-workers retrospectively investigated compound action potentials (CAPs) in 3 adult patients with SNHL or MHL undergoing revision Vibroplasty, and reported CAP thresholds to be suitable for identifying good mechanical FMT coupling [15]. Mandalà and co-authors reported similar observations for RW-Vibroplasty in 14 infants and children suffering from CHL or MHL [16]. Colletti and co-workers prospectively examined 26 adult patients with CHL or MHL undergoing RW-Vibroplasty. The average improvement in postoperative aided air-conduction thresholds after modification of FMT coupling according to intraoperative CAPs was significantly better compared to the control group, where no intraoperative CAPs were measured. (54.68.9 vs. 41.711.1 dB-HL, *p* = 0.0032) [17]. Geiger and colleagues retrospectively evaluated intraoperative auditory brainstem responses (ABRs) to assess FMT coupling-quality with numerous different coupling-modalities in a total of 30 adult patients with mild-to-severe HL. The authors considered the ABRs to be useful in the estimation of FMT coupling-efficacy in all types of Vibroplasty explored [13]. Most recently, Fröhlich and co-workers reported on the effectiveness of ABRs to assess FMT coupling-quality in a diagnostic multicenter study including 23 patients who received a VSB with different coupling-modalities [12]. Intraoperative ABR responses were observed in 22 of 23 patients. In the one patient without ABR data, insufficient coupling of 36.7 dB was postoperatively confirmed. The authors concluded that intraoperatively-measured ABR responses have the potential to predict the VSB’s FMT coupling-quality [12].

In contrast to the number of available studies that assess coupling-quality via intraoperative measurements, studies that explore different methods of fixation of the FMT during Vibroplasty are sparse and only available from retrospective temporal bone experiments: Müller and co-authors retrospectively compared the transmission behavior of various FMT couplers in 32 specimens using laser Doppler vibrometry [18]. The authors observed a tendency towards better coupling-quality with LP couplers at low frequencies (500–1000 Hz) and Clip couplers at high frequencies (2000–4000 Hz). However, both observations missed the significance level (both *p* > 0.05). The authors concluded that the differences in this experimental setting were minimized by individual biasing factors [18].

There are currently no clinical studies in the literature that prospectively explore (a) the influence of intraoperative ABR measurements on coupling-quality compared to a matched-cohort of patients undergoing Vibroplasty without intraoperative ABR measurements. In addition, no clinical studies (b) prospectively explore the influence of various fixation methods of the FMT during RW-Vibroplasty on the coupling-quality. Finally, no clinical studies have prospectively investigated (c) the additional safety intraoperative ABR measurements provide compared to a matched-cohort of patients undergoing Vibroplasty without intraoperative ABR measurements.

In the present work, patients undergoing Vibroplasty were prospectively explored for the influence of intraoperative ABR on postoperative coupling-quality compared to a matched-cohort of patients without intraoperative ABR for postoperative audiologic outcomes, including the FMTs coupling-quality. In addition, the influence of various fixation methods for RW-coupling were explored: (1) cartilage-counter-bearing, (2) cartilage-counter-bearing and cartilage-housing, (3) cartilage-counter-bearing, cartilage-housing and injectable-platelet-rich-fibrin (iPRF), as previously proposed by Beltrame and colleagues [19]. Finally, the possible additional safety provided by intraoperative ABR measurements performed during Vibroplasty compared to matched-cohort patients undergoing Vibroplasty without intraoperative ABR measurements was investigated.

## Materials and methods

### Study population

This prospective pilot study was conducted at ***the Department of Otorhinolaryngology of the Medical University of Innsbruck*** and investigated (a) the influence of intraoperative ABR measurements on coupling-quality if compared to matched-cohort of patients undergoing Vibroplasty without intraoperative ABR measurements, (b) the influence of various fixation methods of the FMT during RW-Vibroplasty on the coupling-quality and (c) the possible additional safety provided by intraoperative ABR measurements performed during Vibroplasty. All eligible patients were prospectively enrolled over a 3-year period. Inclusion criteria were: (1) age ≥ 18 years, (2) moderate to profound sensorineural, conductive or mixed hearing loss, (3) limited satisfaction with previously-worn hearing aids and (4) written informed consent to participate in the study. Exclusion criteria were: (1) age < 18 years, (2) mild sensorineural, conductive or mixed hearing loss or (3) bordering to deafness sensorineural, conductive or mixed hearing loss. All enrolled patients underwent (a) preoperative audiological testing, (b) Vibroplasty with intended RW-coupling, (c) intraoperative ABR measurement and (d) postoperative audiological testing including the assessment of coupling-quality. The study was approved by the local ethics committees (ethics committees’ reference number: 1233/2018). The matched control group was recruited from otologic patients treated at our institution. All patients treated at our institution sign a general informed consent prior to treatment that they agree to the use of collected disease data and findings in the context of scientific studies in pseudonymous form. Thus, a specific written informed consent was not obtained from these patients due to the retrospective inclusion of their routinely generated clinical data.

To compare the efficacy and safety of Vibroplasty with intraoperative ABR, a case-matched cohort was generated from a larger sample of patients who underwent Vibroplasty without intraoperative ABR measurement at ***the Department of Otorhinolaryngology of the Medical University of Innsbruck***. Inclusion criteria for the case-matched cohort were the same as for the study cohort except for availability of intraoperative ABR measurements.

### Audiometric assessment

#### Preoperative sound perception

Preoperative sound perception was tested via pure-tone audiometry the day before surgery with an Interacoustics AC40 audiometer in a sound-treated room according to audiometric standards (ENISO8253 1-3). Air-conduction- and bone-conduction thresholds were measured at audiometric frequencies between 0.125 and 8 kHz. If necessary, the contralateral ear was masked with narrow-band noise signals. The bone-conduction thresholds were defined as the primary preoperative outcome parameter. Preoperative bone-conduction pure-tone-average (BC-PTA3) was calculated as the mean of the thresholds obtained at 1.0 kHz, 2.0 kHz and 4.0 kHz, as previously proposed [12]. Thereby, the VSB’s performance spectrum, with stronger amplification power in the high frequency range, was taken into account [12].

#### Preoperative word recognition

Preoperative word-recognition-scores (WSR) were routinely measured in all patients of the study cohort and case-matched cohort the day before surgery with an Interacoustics AC40 audiometer. WRS was measured using the German “Freiburger” speech-intelligibility test, using 50-word lists of monosyllabic nouns at suprathreshold presentation levels at 100 dB SPL under free-field conditions with masking of the contralateral ear with small-band signal. Results are presented as percentage values.

#### Intraoperative auditory brainstem response measurements

The intraoperative ABR measurements were performed using an Interacoustics Eclipse device as previously described [20, 21]. The system allowed for the generation of the stimulus and the recording of the brainstem potentials. The auditory stimulus was transmitted to the VSB via an interface to the implant (MED-EL AcoustiAP, Innsbruck, Austria) The stimulus generated by the Eclipse ABR device was a chirp stimulus (CE-Chirp^®^) at a stimulation rate of 49.1 Hz. The stimuli were previously calibrated accordingly and thresholds were measured in dB-HL. The ABR signal was recorded through surface electrodes placed on the skin of the left and right mastoid (reference electrodes); the Cz position (active electrode) and the ground electrode was placed on the cheek. The skin was treated to provide impedances of less than 5 kΩ, which was checked once prior to the beginning of the surgery. A total of 2000 stimulus repetitions were presented in one measurement run. The auditory Evoked Potentials (EP) were recoded using a pre-amplifier (EP25, Eclipse, Interacoustics) and the stimuli delivered by a research tool, consisted of an interface box (AcoustiAP, MED-EL, Innsbruck, Austria) directly connected to the EP device via audio cable. An audiologist operated the ABR device and interpreted the ABR signal during the VSB surgery.

#### Postoperative sound perception

Postoperative sound perception was tested via pure-tone audiometry, which was performed the day after surgery as described above for the preoperative test. Due to the ear bandage, only the bone-conduction thresholds were measured at audiometric frequencies between 0.125 and 8 kHz. If necessary, the contralateral ear was again masked with narrow-band noise signals. Bone-conduction hearing thresholds were defined as the primary postoperative outcome parameter. Bone-conduction pure-tone-average (BC-PTA3) was calculated as the mean of the thresholds obtained at 1.0 kHZ, 2.0 kHZ and 4.0 kHZ. For these measurements, the audio-processor of the VSB was not worn.

#### Postoperative assessment of coupling-quality

Postoperative coupling-quality was tested by measuring the threshold of stimulation with VSB directly via the audio processor (Samba 2) using the vibrogram function of the fitting software (Symfit 8.0 MED-EL). The vibrogram measurements were conducted during the first fitting session which took place 4–6 weeks after the surgery. Comparing the bone conduction hearing thresholds with the thresholds obtained from the vibrogram allows the FMT’s coupling-quality to be assessed [12].

#### Postoperative word recognition

Postoperative WSR were routinely measured in all patients of the study cohort and case-matched cohort 4 to 6 weeks after surgery as described above for the preoperative test. The 50-word lists of monosyllabic nouns were presented at suprathreshold presentation levels at 65 dB SPL under free-field conditions with masking of the contralateral ear with small-band signal. In all patients VSB was active and used. Results are presented as percentage values.

### Surgical technique

Surgery was performed by one surgeon (J.S.) with 10 years of experience in AMEI implantation. All procedures were planned for RW-coupling. After obtaining written informed consent, a facial-nerve monitoring device (Neurosign 100, Neurosign surgical, UK) as well as a setup for the ABR measurement as described above was installed and checked for functionality. The surgical approach was planned individually, based on the preoperative finding. For the RW-coupling, a standard RW-coupler was used. The RW-recess was prepared for optimal FMT positioning with a 1.8-mm diamond burr, reducing the bony overhang of the RW and enlarging the hypotympanon to properly fit the 1.8 × 2.3 FMT. Afterwards, the FMT was put in place in three different steps, according to our clinic’s established standard procedure with (1) cartilage-counter-bearing, (2) cartilage-counter-bearing and cartilage-housing and (3) iPRF fixation. This has previously been reported on by Beltrame and colleagues in 2014 [19]. All three coupling techniques were measured in each patient of the study group.

Intraoperative pictures of the three consecutive, different fixation methods for RW-coupling during Vibroplasty as previously proposed by Beltrame and colleagues [19]. A depicts cartilage-counter-bearing, B depicts cartilage-counter-bearing and cartilage-housing and C depicts cartilage-housing with cartilage-housing and iPRF. For additional detail, please refer to the main text.

**Fig. 1:**
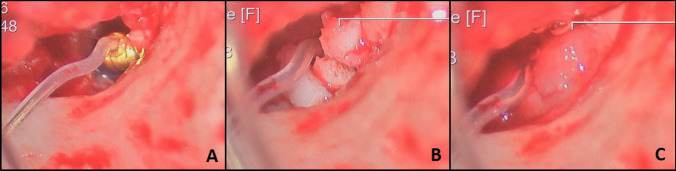
Three consecutive, different fixation methods for round-window-coupling during Vibroplasty as previously proposed by Beltrame and colleagues [19]. Panel **A** shows the positioned FMT at the round window with the cartilage-counter-bearing. Panel **B** depicts the applied cartilage-housing and **C** visualizes the situation after application of the iPRF

The following fixation methods for RW-coupling were explored: (1) cartilage-counter-bearing, (2) cartilage-counter-bearing and cartilage-housing, (3) cartilage-counter-bearing, cartilage-housing and injectable-platelet-rich-fibrin (iPRF; Fig. 1), as previously proposed by Beltrame and colleagues [19]

The patients with the two different fixation methods were primary planned for RW-coupling. This approach had to be aborted intraoperatively due to the surgical situation and patient’s anatomy. OW-coupling was performed in a subject with atresia who had an unpredicted course of the facial nerve blocking the access to the RW-niche. The stapes-coupling occurred due to a subtotal ossification of the RW and a usable stapes superstructure. The modification of the coupling-site was necessary to reduce the risk of inner ear damage and was performed with a standard CliP coupler.

### Statistical analysis

#### Calculation of outcome parameters

Pre- and postoperative BC-PTA, postoperative vibrograms and postoperative coupling-quality were calculated as the mean of bone conduction hearing thresholds as well as vibrogram values at 1-, 2-, and 4 kHz. Intraoperative ABR measurements were reported as corresponding hearing thresholds in dB-HL. Postoperative coupling-quality was calculated by subtracting the postoperative BC-PTA3 from postoperative Vibrogramm-PTA3, as previously proposed [14].

#### Propensity score matching

To explore the influence of intraoperative ABR measurements on the FMTs’ coupling-quality, a case-matched cohort was generated from a larger sample of patients who underwent Vibroplasty without intraoperative ABR at ***the Department of Otorhinolaryngology of the Medical University of Innsbruck***. A propensity-score-matching plug-in for SPSS24 (IBM, Armonk, NY) was used. The case-matched cohort was matched to the study cohort for (1) preoperative BC-PTA3 with a maximum difference of <  ± 10 dB-HL as acceptable tolerance margin for possible matches, (2) type of hearing loss, (3) coupling-site of FMT, (4) sex, (5) age and (6) number of previous surgeries.

#### Outcome parameters

The main outcome parameters of the analysis were (1) “mean postoperative coupling-quality PTA3”, to explore the influence of intraoperative ABR measurements during Vibroplasty on the postoperative coupling-quality, (2) “mean intraoperative ABR threshold”, to assess the influence of various fixation methods for RW-coupling, including: (a) cartilage-counter-bearing, (b) cartilage-counter-bearing and cartilage-housing and (c) cartilage-counter-bearing, cartilage-housing and iPRF on coupling-quality and (3) “postoperative BC-PTA3”, to explore the influence of intraoperative ABR measurements on Vibroplasty safety. Both (1) mean postoperative coupling-quality PTA3 and (3) postoperative BC-PTA3 were compared to matched-cohort patients undergoing Vibroplasty without intraoperative ABR. In addition, preoperative WRS presented at suprathreshold presentation levels at 100 dB SPL and postoperative WRS presented at suprathreshold presentation levels of 65 dB SPL with VSB active and used are provided.

### Statistical analysis

For continuous data, means and standard deviations (SD) as well as minimums and maximums were calculated. Mann–Whitney-*U* and Wilcoxon tests were used to test for statistical significance defined as *p* < 0.05. Additionally, repeated-measure ANOVA analyses were performed. Bonferroni correction was applied to multiple comparisons. All calculations were performed with SPSS24 and its corresponding plug-ins.

## Results

### Study population

A total of 14 patients (6 female, 8 male) with moderate-to-profound sensorineural, conductive or mixed hearing loss and limited satisfaction with previously worn hearing aids were prospectively included in a three-year study at ***the Department of Otorhinolaryngology of the Medical University of Innsbruck***. Mean age was 51 (± 9) years, ranging from 22 to 71 years. Four patients suffered from conductive hearing loss and 10 from mixed hearing loss. The mean number of previous surgeries was 2 (± 1), ranging from 0 to 3 previous surgeries. Mean preoperative BC-PTA3 was 40.59 (± 16.19) dB-HL, ranging from 20.00 to 65.00 dB-HL. Mean preoperative WRS at 100 dB SPL was 71% (± 21%), ranging from 30 to 100%. All procedures were intended for RW-coupling. Due to intraoperative inability to visualize the RW, OW-coupling was performed in one patient and stapes-superstructure-coupling in another patient.

From a total of 62 patients undergoing Vibroplasty without intraoperative ABR measurements, a cohort of 14 patients was matched based on (1) preoperative BC-PTA3 with a maximum difference of <  ± 10 dB-HL as acceptable tolerance margin for possible matches, (2) type of hearing loss, (3) coupling-site of FMT, (4) sex, (5) age and (6) number of previous surgeries using the Propensity-score-matching plug-in for SPSS24 (IBM, Armonk, NY). Of these, 8 patients were female. Mean age was 58 years (± 15) years, ranging from 30 to 84 years. Four patients suffered from conductive hearing loss and 10 from mixed hearing loss. The mean number of previous surgeries was 2 (± 1), ranging from 0 to 4 pervious surgeries. Mean preoperative BC-PTA3 was 40.60 (± 14.32) dB-HL, ranging from 16.67 to 61.67 dB-HL. Mean preoperative WRS at 100 dB SPL was 75% (± 15%), ranging from 45 to 100%. RW-coupling was performed in 13 patients, and stapes-superstructure-coupling in one patient.

Predefined preoperative matching criteria ensured no significant differences between the study cohort and the case-matched cohort (all *p* > 0.301). In addition, the mean preoperative WRS at 100 dB SPL did not significantly differ between the study cohort and the case-matched cohort (*p* = 0.927). The single study patient undergoing OW-Vibroplasty was matched to a cohort patient undergoing RW-Vibroplasty, since no other cohort patient undergoing OW-Vibroplasty was available who met the other predefined matching criteria. Detailed data of all 28 patients included in the statistical analysis are presented in Table 1.

**Table 1:** Clinical data of all 14 study patients and all 14 cohort-matched patients^##^

	With intraoperative ABR	Without intraoperative ABR	*p* value
**Sex**			
Male	8	6	0.706*
Female	6	8	
**Mean age (years)**	51 (± 15; 22–71)***	58 (± 15; 30–84)***	0.301**
**Surgical site**			
Left	7	7	1.000*
Right	7	7	
**Type of hearing loss**			
Conductive hearing loss	4	4	1.000*
Mixed hearing loss	10	10	
**Number of previous surgeries**	2 (± 1; 0–3)***	2 (± 1;0–4)***	0.454*
**Coupling-site**			
Oval window^**#**^	1	0	0.595*
Round window	12	13	
Stapes suprastructure	1	1	
**Surgeon**			
***JS***	14	14	1.000*
**Mean preoperative BC PTA3**	40.59 (± 16.19; 20.00–65.00)***	40.60 (± 14.32; 16.67–61.67)***	0.534**

^*^Chi-squared test; **Mann–Whitney-*U*-Test; ***Standard deviation & range; ^**#**^Only one patient underwent oval-window-coupling and was therefore matched with a patient who had round-window-coupling; ^**##**^Matching of study patients that underwent intraoperative ABR with control patients who did not undergo intraoperative ABR based on (1) preoperative BC PTA3 with a difference lower than 10 dB-HL, (2) type of hearing loss, (3) coupling-site of the FMT, (4) sex, (5) age and (6) number of previous surgeries

### Outcome parameters

#### Influence of intraoperative ABR measurements on the postoperative coupling-quality

Mean postoperative coupling-quality was 1.79 (± 6.80) dB-HL, ranging from − 16.67 to + 13.33 dB-HL for patients undergoing Vibroplasty with intraoperative ABR compared to 12.26 (± 12.60) dB-HL, ranging from − 5.00 to + 43.33 dB-HL for matched patients undergoing Vibroplasty without intraoperative ABR. This difference was significant (Mann–Whitney-*U*-Test; *p* = 0.006; Table 2).

**Table 2:** Influence of intraoperative ABR measurements on the postoperative coupling-quality

	With intraoperative ABR	Without intraoperative ABR	*P* value
Mean postoperative coupling-quality PTA3	1.79 (6.80; − 16.67–13.33)**	12.26 (− 12.60; − 5.00–43.33)**	0.006*
Mean postoperative Vibrogram PTA3	43.21 (18.26; 23.75–63.33)**	54.05 (15.74; 26.67–78.33)**	0.161*

^*^Mann–Whitney-*U*-Test; **Standard deviation & range; negative values indicate an improvement of BCA PTA3, positive values indicate a deterioration of BCA PTA3

Mean postoperative Vibrogram PTA3 was 43.21 (± 18.26) dB-HL, ranging from 23.75 to 63.33 dB-HL for patients undergoing Vibroplasty with intraoperative ABR, compared to 54.05 (± 15.74) dB-HL, ranging from 26.67 to 78.33 dB-HL for matched patients undergoing Vibroplasty without intraoperative ABR. This difference was not significant (Mann–Whitney-*U*-Test; *p* = 0.161; Table 2).

#### Influence of various fixation methods for RW-coupling on coupling-quality

For RW-Vibroplasty with cartilage-counter-bearing, the mean intraoperative ABR threshold was 56 (± 12) dB-HL, ranging from 30 to 70 dB-HL. For RW-Vibroplasty with cartilage-counter-bearing and cartilage-housing, the mean intraoperative ABR threshold was 53 (± 11) dB-HL, ranging from 30 to 70 dB-HL. For RW-Vibroplasty with cartilage-counter-bearing, cartilage-housing and iPRF, the mean intraoperative ABR threshold was 57 (± 12) dB-HL, ranging from 35 to 75 dB-HL (Table 3).

**Table 3:** Influence of various fixation methods for RW-coupling on coupling-quality

	Mean ABR threshold (dB-HL)	*p*-value*	Partial eta square**
Cartilage-counter-bearing	56 (12; 30–70)	0.141	n.a
Cartilage-counter-bearing and cartilage-housing	53 (11; 30–70)	0.039	0.332**
Cartilage-counter-bearing, cartilage-housing and iPRF fixation	57 (12; 35–75)	0.556	n.a

^*^Bonferroni corrected Friedman-test for related samples; **partial eta square > 0.16 are considered as strong effect. Intraoperative re-adjustment of FMT was performed in one patient (7% of the patients); *iPRF* injectable-platelet-rich-fibrin

Bonferroni corrected Friedman-test for related samples revealed that the mean intraoperative ABR threshold for RW-Vibroplasty with cartilage-counter-bearing and cartilage-housing significantly differed from the other two fixation methods, with a strong effect size (Bonferroni corrected Friedman-test; *p* = 0.039; partial eta square 0.332; Table 3).

Intraoperative re-adjustment of the FMT was performed in one patient (7% of the study patients), based on an unsatisfactory intraoperative ABR threshold of 80 dB-HL. After three additional re-adjustments, the intraoperative ABR threshold improved from 80 to 70, 70 and 50 dB-HL, respectively. In this study, a satisfying intraoperative ABR threshold was defined in the average range of the preoperative bone-conduction. In this specific patient, the preoperative BC-PTA3 averaged around 50 dB-HL.

#### Influence of intraoperative ABR measurements on Vibroplasty safety

**Fig. 2:**
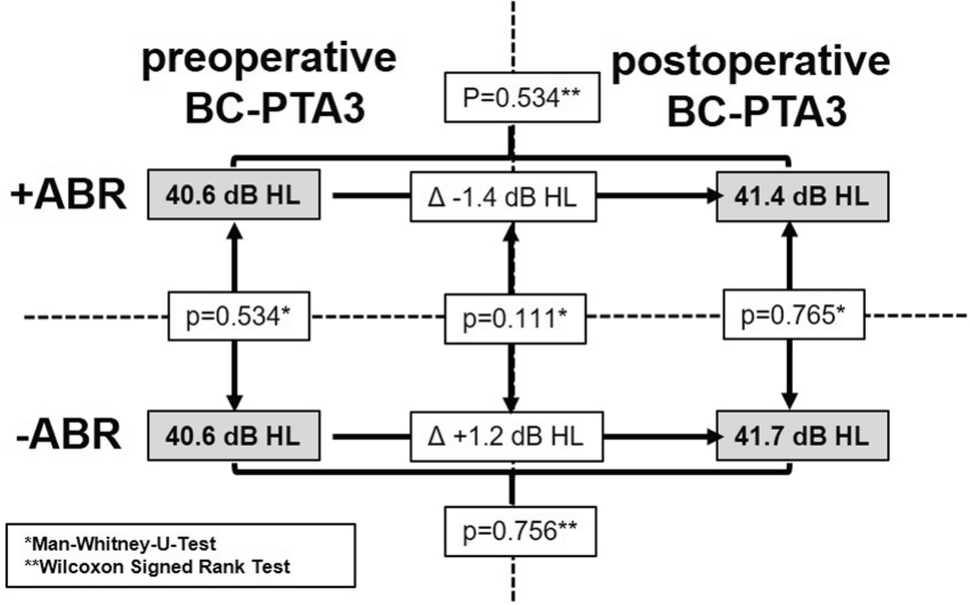
Influence of intraoperative ABR measurements on Vibroplasty safety. Mean preoperative bone-conduction (BC; left side of the diagram) and mean postoperative BC—(right side of the diagram) pure-tone averages (PTA3). Patients undergoing Vibroplasty with intraoperative auditory brainstem response (ABR) measurements are depicted in the upper part of the diagram (+ ABR); patients undergoing Vibroplasty without intraoperative ABR measurements are depicted in the lower part of the diagram (-ABR). For standard deviations and ranges, refer to the main text. One of the primary cohort-matching criteria was the preoperative BC-PTA3, which did not significantly differ between study patients and matched-cohort patients (40.6 vs. 40.6 dB-HL, Man-Whitney-*U*-Test; *p* = 0.534), suggesting successful Propensity-Score-Matching. Postoperative BC-PTA3 did not significantly differ between study patients and matched-cohort patients (41.4 vs. 41.7 dB-HL; Man-Whitney-*U*-Test; *p* = 0.765), suggesting no additional safety benefit of intraoperative ABR measurements. In addition, the change between pre- and postoperative BC-PTA3 did not differ significantly (− 1.4 vs. + 1.2 dB HL; Wilcoxon Signed Rank Test; *p* = 0.111)

Mean postoperative BC-PTA3 was 41.43 (± 14.96) dB-HL, ranging from 16.67 to 60.00 dB-HL for patients undergoing Vibroplasty with intraoperative ABR, compared to 41.79 (± 18.37) dB-HL, ranging from 8.33 to 61.67 dB-HL for matched patients undergoing Vibroplasty without intraoperative ABR. This difference was not significant (Mann–Whitney-*U*-Test; *p* = 0.765; Table 4, Fig. 2).

**Table 4:** Influence of intraoperative ABR measurements on Vibroplasty safety

	With intraoperative ABR	Without intraoperative ABR	*P* value
Mean postoperative BC PTA3	41.43 (14.96; 16.67–60.00)**	41.79 (18.37; 8.33–61.67)**	0.765*
Mean Delta preoperative vs. postoperative BC PTA3	− 1.43^**#**^ (11.47; − 18.33–30.00)	+ 1.19^#^ (5.89; − 11.67–8.33)	0.111*

^*^Mann–Whitney-*U*-Test; **Standard deviation & range; **#**negative values indicate an improvement of BCA PTA3; positive values indicate a deterioration in BCA PTA3

Mean postoperative change between preoperative- and postoperative BC-PTA3 was − 1.43 (± 11.47) dB-HL, ranging from − 18.33 to + 30.00 dB-HL for patients undergoing Vibroplasty with intraoperative ABR, compared to + 1.19 (± 5.89) dB-HL, ranging from – 11.67 to + 8.33 dB-HL. This difference was not significant (Mann–Whitney-*U*-Test; *p* = 0.111; Table 4, Fig. 2). Here, positive dB-HL values indicate a deterioration of BC-PTA3.

#### Influence of intraoperative ABR measurements on postoperative sound perception

Mean postoperative WRS at 65 dB SPL was 75% (± 16%), ranging from 45% to 100.00% for patients undergoing Vibroplasty with intraoperative ABR, compared to 60% (± 20%), ranging from 30 to 80%. This difference was significant (Mann–Whitney-*U*-Test; *p* = 0.028).

## Discussion

AMEIs are currently considered an appropriate treatment alternative for patients with moderate to profound hearing loss who are unable to wear HAs [1, 2]. In such patients, the VSB, due to its considerable variety of possible coupling-sites, is often considered to be the AMEI of choice [3–10]. Sufficient transfer of the FMT’s energy to the inner ear is a crucial factor for proper function and is referred to as “coupling-quality” [11]. While various, mainly retrospective studies that intraoperatively measure coupling-quality have been published [12–17], studies exploring the influence of different methods of FMT fixation are sparse and only available from retrospective temporal bone experiments [18].

This study prospectively explored the influence of intraoperative ABR measurements on coupling-quality compared to a matched-patient cohort who underwent Vibroplasty without intraoperative ABR. Additionally, we explored the effect on coupling-quality of the various fixation possibilities of the FMT during RW-Vibroplasty. Finally, we evaluated the contribution of intraoperative ABR measurements on the safety of the procedure. The present study aims to close this gap by providing necessary data.

The first key finding of the present study was that intraoperative ABR measurements appear to result in significantly better postoperative coupling-quality when compared to matched-cohort patients. While the mean postoperative coupling-quality observed for the matched-patient cohort undergoing Vibroplasty without intraoperative ABR measurements was 12.26 dB-HL, it was 1.79 for study subjects (Mann–Whitney-*U*-Test; *p* = 0.006; Table 2). Thus, additional intraoperative ABR measurements, which extend the duration of Vibroplasty surgery for approximately 10 to 20 min, appear to ultimately result in significantly better postoperative audiological performance.

Although no mean postoperative coupling-quality was provided, similar observations were reported by Fröhlich and colleagues, with narrow distribution of postoperative coupling- qualities between 0 and 15 dB for 18 or 23 patients in Bland–Altman plots [12]. In addition, Müller and co-authors observed stable coupling-qualities of 12.7 dB-HL approximately 6 months after RW-Vibroplasty without intraoperative ABR measurements [18]. A loss of approximately 12 dB-HL in Vibroplasty without intraoperative ABR measurement as observed in our study control group correlates with the literature. An improvement of 10 dB-HL as reported above can be seen as an improvement which optimizes RW-Vibroplasty.

The second key finding of the present study was that RW-Vibroplasty with cartilage-counter-bearing alone was observed to result in a mean ABR threshold of 56 dB. The intraoperative measurements showed a further improvement to 53 dB if a cartilage-housing is used. Unfortunately, the addition iPRF reversed this gain to a mean of 57 dB. Comparing these observed changes, the usage of a positioned cartilage-counter-bearing with cartilage-housing showed a significant advantage for the coupling-quality (Bonferroni corrected Friedman-test; *p* = 0.039; Table 3). The addition of iPRF to further fixate the construction leads to an immediate decline in the coupling-quality. Although this observation might appear counterintuitive at first, the intraoperative iPRF fixation appeared to soften both the cartilage-counter-bearing and cartilage-housing. This significant difference in intraoperative ABR threshold had a strong effect size (partial-eta-square = 0.332; Table 3). Whether or not omitting the iPRF fixation leads to further improvement in the overall long-term Vibroplasty coupling-quality needs to be evaluated in future studies. The assumption that the missing iPRF fixation results in less stability and long-term functional loss is still subject to debate.

The fixation technique tested above was published by Beltrame and co-authors in 2014 [19]. The knowledge achieved in the present study was not available at the time. The authors strongly encourage a prospective study evaluating the value of omitting injectable iPRF in RW-Vibroplasty to see if it results in a decline in long-term stability.

Unfortunately, there are no other studies exploring the influence of various fixation methods in RW-Vibroplasty available. Fröhlich and colleagues reported a mean intraoperative ABR threshold of 30 dB-HL for RW-Vibroplasty including RW-soft-coupling and RW-direct-coupling [12]. The intraoperative ABR threshold observed in the present study is considerably higher than reported by Fröhich and co-workers [12]. However, their study included a total of only five patients with RW-Vibroplasty, of which only one patient received cartilage-counter-bearing. This specific patient had, 5 dB-HL. This statistical outlier as well as the comparatively small group of patients (5 patients in the cited study vs. 14 in our study) might provide a possible explanation for the observed difference in intraoperative ABR thresholds [12].

The third and final key finding of the present study was that intraoperative ABR measurements were not observed to improve the safety of Vibroplasty. Mean postoperative BC-PTA3 was 41.43 for study patients undergoing Vibroplasty with intraoperative ABR, compared to 41.79 dB-HL for matched-cohort patients undergoing Vibroplasty without intraoperative ABR (Mann–Whitney-*U*-Test; *p* = 0.765; Table 4, Fig. 2). One possible conclusion to draw from this is that Vibroplasty itself is a complex yet safe and effective surgical procedure, as previously described in other studies [11, 22, 23]. A similar mean postoperative BC-PTA4 of 38 dB-HL was reported by Fröhlich and colleagues [12].

Although performing intraoperative ABR measurements during Vibroplasty did not seem to provide an additional safety benefit, it appeared to increase postoperative audiological performance via improved coupling-quality. In addition, intraoperative ABR measurements might indirectly improve the safety of the procedure itself. One of the 14 patients initially had an unsatisfactory intraoperative ABR threshold of 80 dB-HL. After three additional adjustments of the FMT, the patient’s intraoperative ABR threshold ultimately improved to 50 dB-HL. Thus, this specific patient was spared revision Vibroplasty surgery and all the general and specific risks it can entail. Although one out of 14 patients might appear insignificant, this translates to an indirect safety benefit of approximately 7% of the patients who had intraoperative ABR measurements and did not need revision surgery.

Finally, performing intraoperative ABR measurements during Vibroplasty appeared to result in a significantly better mean postoperative WRS at 65 dB-SPL. For patients undergoing intraoperative ABR measurements during Vibroplasty mean postoperative WRS at 65 dB-SPL was observed at 75% as compared to 60% for patients undergoing Vibroplasty without intraoperative ABR measurements. This postoperative WRS at 65 dB-SPL after Vibroplasty for the group of study-patients is in range with previous observations, reporting postoperative WRS from 67.1% to 72.6% [24, 25]. In one of the patients of the study group, pre- and postoperative WRS could not be obtained, since this single patient was not a German native-speaker.

Some limitations of the present study need to be addressed. Although this study was prospectively performed, the control group consisted of a retrospectively-matched cohort (Table 1). A prospective study that directly compares patients undergoing Vibroplasty with and without intraoperative ABR measurements would provide more reliable data. Secondly, the number of patients included in the study was small. Larger, prospective studies exploring this specific scientific question should be conducted. In addition, word recognition was assessed in the present study. However, sound pressure levels for the WRS were 100 dB-SPL before surgery and 65 dB-SPL after surgery. Consequently, a reliable comparison between pre- and postoperative WRS was not feasible. The higher SPL for preoperative WRS was chosen to assess a remaining word recognition ability, which can possibly technically amplified. Follow-up studies exploring the postoperative word recognition score for patients undergoing Vibroplasty with and without intraoperative ABR measurement should be performed. Finally, we intended all included study-patients to undergo RW-Vibroplasty. However, in two patients the RW could not be visualized intraoperatively. In one patient, OW-Vibroplasty was performed instead; the other patient received stapes-superstructure-coupling. These two different coupling-techniques were included in the total number of patients to obtain a larger study population.

## Conclusion

Intraoperative ABR measurement in patients undergoing Vibroplasty was observed to improve postoperative coupling-quality, compared to matched-cohort patients without intraoperative ABR measurement. Moreover, the fixation of the FMT with cartilage-counter-bearing and cartilage-housing appeared to result in a superior coupling-quality, while the addition of iPRF reduced the coupling-quality. Finally, we observed an additional safety benefit for study patients undergoing Vibroplasty with intraoperative ABR. The FMT was readjusted in 7% of the study patients based on an unsatisfactory intraoperative ABR threshold. Thus, intraoperative ABR measurements may provide indirect safety benefits by sparing these patients revision Vibroplasty surgery due to undetected insufficient coupling-quality.

## Data Availability

The data will be available on request to the corresponding author.

## Abbreviations

BC: Bone-conductive
CHL: Conductive hearing loss
dB: Decibel
dB-HL: Decibel hearing level
FMT: Floating-mass-transducer
HA: Hearing aid
iPRF: Injectable platelet rich fibrin
kHz: Kilo hertz
MHL: Mixed hearing loss
PTA: Pure tone average
SNHL: Sensorineural hearing loss
VSB: Vibrant Soundbridge^®^
