# Correction: The influence of intraoperative auditory brainstem responses on vibroplasty coupling-quality and analysis of the impact of different fixation steps on the coupling

**DOI:** 10.1007/s00405-023-08154-y

**Published:** 2023-08-03

**Authors:** Daniel Dejaco, David Riedl, Timo Maria Gottfried, Matthias Santer, Annette Runge, Josef Seebacher, Philipp Zelger, Lia Bicego, Joachim Schmutzhard

**Affiliations:** 1 Department of Otorhinolaryngology—Head and Neck Surgery, Medical University of Innsbruck, Anichstr. 35, 6020 Innsbruck, Austria; 2 Department of Medical Psychology, Medical University of Innsbruck, Schöpfstr. 23a, 6020 Innsbruck, Austria; 3 Department for Hearing, Speech and Voice Disorders, Medical University of Innsbruck, Anichstr. 35, 6020 Innsbruck, Austria; 4 MED-EL Elektromedizinische Geräte G.M.B.H., Fürstenweg 77a, 6020 Innsbruck, Austria

**Correction: European Archives of Oto-Rhino-Laryngology** 10.1007/s00405-023-08103-9

In the original publication of the article, the following author’s “Lia Bicego” and “Joachim Schmutzhard” first and last names were swapped incorrectly. The names are processed correctly in this article.

